# Enhancing angular photonic spin Hall effect at surface plasmon resonance

**DOI:** 10.1515/nanoph-2025-0206

**Published:** 2025-09-08

**Authors:** Cherrie May Olaya, Norihiko Hayazawa, Maria Herminia Balgos, Takuo Tanaka

**Affiliations:** Metaphotonics Research Team, RIKEN Center for Advanced Photonics, Saitama, 351-0198, Japan; Surface and Interface Science Laboratory, RIKEN Cluster for Pioneering Research, Saitama, 351-0198, Japan; Center for Quantum Conversion Research, Institute for Basic Science, Gwangju, Republic of Korea

**Keywords:** photonic spin Hall effect, surface plasmon resonance, Imbert–Fedorov shift, spin-optics

## Abstract

The photonic spin Hall effect (PSHE) is the deep subwavelength spin component shift of light induced by the spin–orbit interaction of photons. Here, we demonstrate a polarimetric scheme to directly measure the surface plasmon resonance-enhanced angular PSHE in the Kretschmann configuration using a gold film. In contrast to the weak measurement scheme that indirectly measures the spatial term-dominated PSHE using a well-collimated source, we focused the incident beam to a small beam waist and significantly enhanced the angular PSHE. Imbert–Fedorov shift manifested as a displacement offset of the reflected beam, have been taken into account to extract only the PSHE shift. In practical measurements, accounting for this shift enables accurate separation of PSHE from polarization-induced artifacts. Measuring PSHE provides an additional spin degree of freedom, enabling an innovative approach toward spin-controlled nanophotonic applications, including optical sensing, precision metrology, and high-contrast microscopy.

## 1 Introduction

The interaction between a finite-width optical beam and a planar interface results in an apparent shift in the direction of the reflected beam with respect to the optical axis. Displacements along the optical axis are called Goos–Hänchen (GH) shifts, while displacements transverse the optical axis are called Imbert–Fedorov (IF) shifts [1], [2]. While GH shifts originate from the dispersion of reflection coefficients [3], IF shifts originate from the spin–orbit interaction (SOI) of photons due to the conservation of total angular momentum of light [4], [5], [6]. IF shifts are typically observed under circularly polarized incidence and 45°-linearly polarized incidence, which correspond to the eigenmodes of the shift [1]. Interestingly, IF shifts allow the observation of the photonic spin Hall effect (PSHE), which is the splitting of a linearly polarized beam into left (LCP) and right (RCP) circularly polarized spin components [4], [7], [8]. The SOI of the bounded beam at the interface induces a transverse shift of the spin components in opposite directions. The additional spin degree of freedom measured from PSHE is promising for different applications such as precision metrology [9], [10], optical sensing [11], [12], and spin-based nanophotonic devices [13]. Spin component shifts in PSHE are typically just within the nanometer scale, and experimental works thus far have relied on weak measurements to indirectly measure PSHE [14]. Moreover, the measured values mainly depend on the pre- and postselected polarization states, making experiments using weak measurement complex and potential applications limited [15]. To maximize and extend its applications, direct measurement of large PSHE is necessary. Different materials were proposed to provide enhanced PSHE such as dielectrics at Brewster angle [16], anisotropic nanostructures [17], [18], and thin films at surface plasmon resonance (SPR) [19], [20], [21]. Specifically for thin films at SPR, measurement of enhanced PSHE has been proposed for high sensitivity optical sensing [21], [22], [23], [24], [25]. These works, however, are mostly theoretical, and to the best of our knowledge, no experimental measurement has demonstrated the enhancement of PSHE at SPR.

In this work, we experimentally demonstrate the direct measurement of PSHE enhanced at SPR, without using weak measurements. Similar to GH and IF shifts, PSHE also has spatial and angular terms [26], [27], [28], [29]. Majority of reports, however, only focus on the spatial term since incident beams are assumed to be well-collimated, hence disregarding the effect of the incident beam waist. In this work, we enhanced PSHE by exploiting the beam propagation dependence of the angular PSHE term. In practical implementations, however, polarization deviations can introduce an additional IF shift, which could obscure the actual PSHE spin component splitting [30], [31]. Separation of this IF contribution is necessary to accurately quantify PSHE shifts. This work also address this separation.

We begin our discussion by first elucidating analytically the effects of the incident beam waist and how it enhances the angular PSHE. Then, we demonstrate the enhanced PSHE experimentally by considering a polarimetric-based measurement setup to separate the spin components. This scheme would allow for quantitative determination of the absolute shift, thus no longer requiring the use of weak measurements.

## 2 PSHE with focused incident beam

The PSHE manifests as a transverse shift of each spin component, which is determined from the location of the reflected beam centroid of each spin component (Supplementary Section 1). At any given plane in the reflected field *z*_*r*_, the total transverse displacement Γ_±_ can be separated into nonpropagating and propagating terms [1], [28] such that Γ_±_ = *δ*_±_ + *z*_*r*_Θ_±_, where *δ*_±_ and Θ_±_ are the spatial and angular terms of PSHE, respectively. The subscript, ±, corresponds to the left and right spin states or the LCP and RCP components, respectively. Under *p*-polarized incidence, the spatial and angular terms are given by

(1) δ±H=∓k0w02rp2Re1+rsrpcot⁡θk0w02rp2+∂rp∂θ2+rp+rscot⁡θ2,

(2) Θ±H=±2rp2Im1+rsrpcot⁡θk0w02rp2+∂rp∂θ2+rp+rscot⁡θ2,

where *k*_0_ is the wavenumber in free space, *w*_0_ is the minimum beam waist of the incident beam, and *r*_*A*_ with $A\in \left\{p,s\right\}$ is the Fresnel reflection coefficient for the *p* and *s* polarization states. *H* and *V* denote *p* and *s* polarization of a bounded beam, which indicates horizontal and perpendicular polarization with respect to the plane of incidence, respectively. To satisfy transversality conditions, the noncentral wave vector component of a *p*-polarized incident bounded beam contains a tiny *s*-polarized component such that |*H*⟩ = |*p*⟩ − *k*_*y*_ cot*θ*_*i*_|*s*⟩. Similarly, for the *s*-polarized incident bounded beam, the polarization is |*V*⟩ = |*s*⟩ + *k*_*y*_ cot*θ*_*i*_|*p*⟩. Here, |*p*⟩ and |*s*⟩ correspond to the *p* and *s* plane wave, and the out-of-plane wave vector spread *k*_*y*_ is responsible for PSHE [14]. Eqs. (1) and (2) account the noncentral wave vector components by considering up to the first order approximation of the Taylor series expansion at *k*_*ix*_ of *r*_*p*_ and *r*_*s*_, which give rise to the second and third terms in the denominator. The second term corresponds to the in-plane spread of the wave vectors, and the third term corresponds to the out-of-plane spread of the wave vectors. Should the incident beam be treated roughly as a plane wave, i.e., only the zeroth order approximation is used for *r*_*p*_ and *r*_*s*_ and $\cot^{2}\theta \ll k_{0}^{2}w_{0}^{2}$ , Eqs. (1) and (2) will degenerate in the transverse shifts derived in Ref. [21], which has been typically used for theoretical considerations of PSHE. While these theoretical considerations are generally sufficient, Refs. [29], [32], [33] have shown that wave vector spread affects the values around the Brewster angle when an air–dielectric interface is used. In this work, we show that this effect extends to materials that exhibit sudden changes in the Fresnel coefficients such as when exciting surface plasmons at resonance.

Both amplitude and phase of *r*_*p*_ and *r*_*s*_ have a large difference around the SPR region, which induces strong SOI. The real factor in Eq. (1) is dominated by the amplitude term of the Fresnel coefficients. As such, the sharp dip in reflectivity at SPR would induce a sharp peak in $\delta_{\pm }^{H}$ . The imaginary term in Eq. (2), on the other hand, is dominated by the phase difference between *r*_*s*_ and *r*_*p*_. The sharp phase change at SPR under *p*-polarized incidence would thus induce a large $\Theta_{\pm }^{H}$ at the vicinity of the SPR angle.

**Figure 1:**
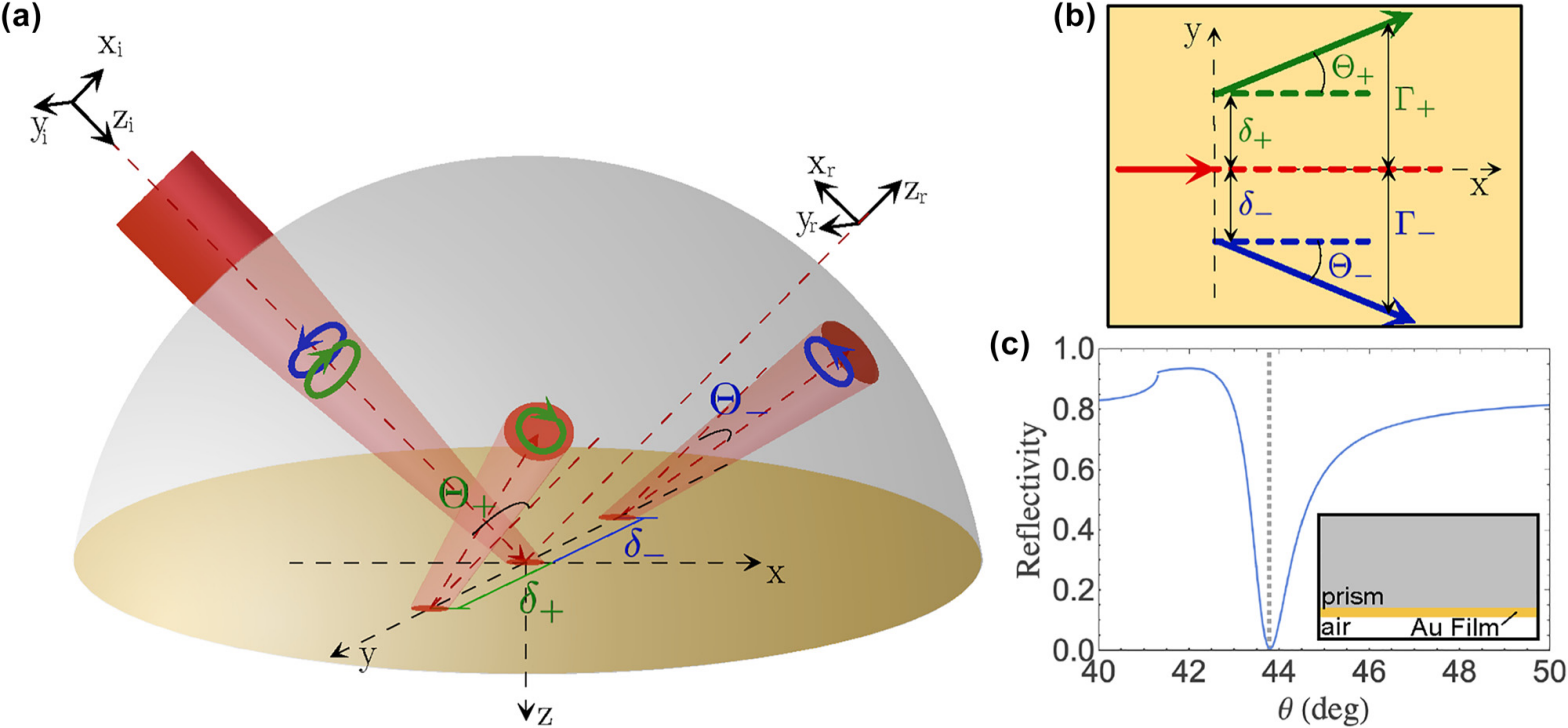
Schematic diagram of the transverse shift in (a) three- and (b) two-dimensional views. The diagram shown in (b) is viewed from the +*z*-axis. Here, *δ*_±_ and Θ_±_ are the spatial and angular PSHE components, respectively. The red solid arrow corresponds to the incident beam propagating along the incident plane. The red dashed arrow corresponds to the reflected beam should the prediction of geometric optics is considered. The green and blue arrows correspond to the reflected LCP and RCP components, respectively. (c) Reflectivity plot around the SPR region when a plane-wave *p*-polarized incidence is considered shows a sharp dip at the SPR angle (*θ*_*SPR*_ = 43.79°). The structure used in the calculation is shown in the inset.

Calculations of PSHE at SPR were performed by considering the excitation of surface plasmons using the Kretschmann configuration and accounting for focused light incidence. As shown in Figure 1(a), we considered the Cartesian coordinate (*x*, *y*, *z*) with the *z* axis normal to the interface at *z* = 0. We also considered the incident coordinate (*x*_*i*_, *y*_*i*_, *z*_*i*_) and the reflection coordinate (*x*_*r*_, *y*_*r*_, *z*_*r*_) for the incident and the reflected fields, respectively. A two-dimensional schematic of the shift as viewed from the +*z*-axis is also shown in Figure 1(b). Here, we used a linearly polarized 633-nm excitation source propagating through a BK7 prism (*n*_prism_ = 1.515) impinging a 50-nm thick Au film (*ɛ*_*Au*_ = −11.740 + 1.2611*i* [34]) with the other side exposed to air (*n*_*air*_ = 1). Upon reflection, the incident beam (red arrow) splits into its LCP (green) and RCP (blue) components. The nonpropagating *δ*_±_ term is shown in dashed line, and the propagating Θ_±_ term is shown in solid arrow line. The reflectivity plot corresponding to this structure is in Figure 1(c) with the SPR angle located at *θ*_*SPR*_ = 43.79°.

**Figure 2:**
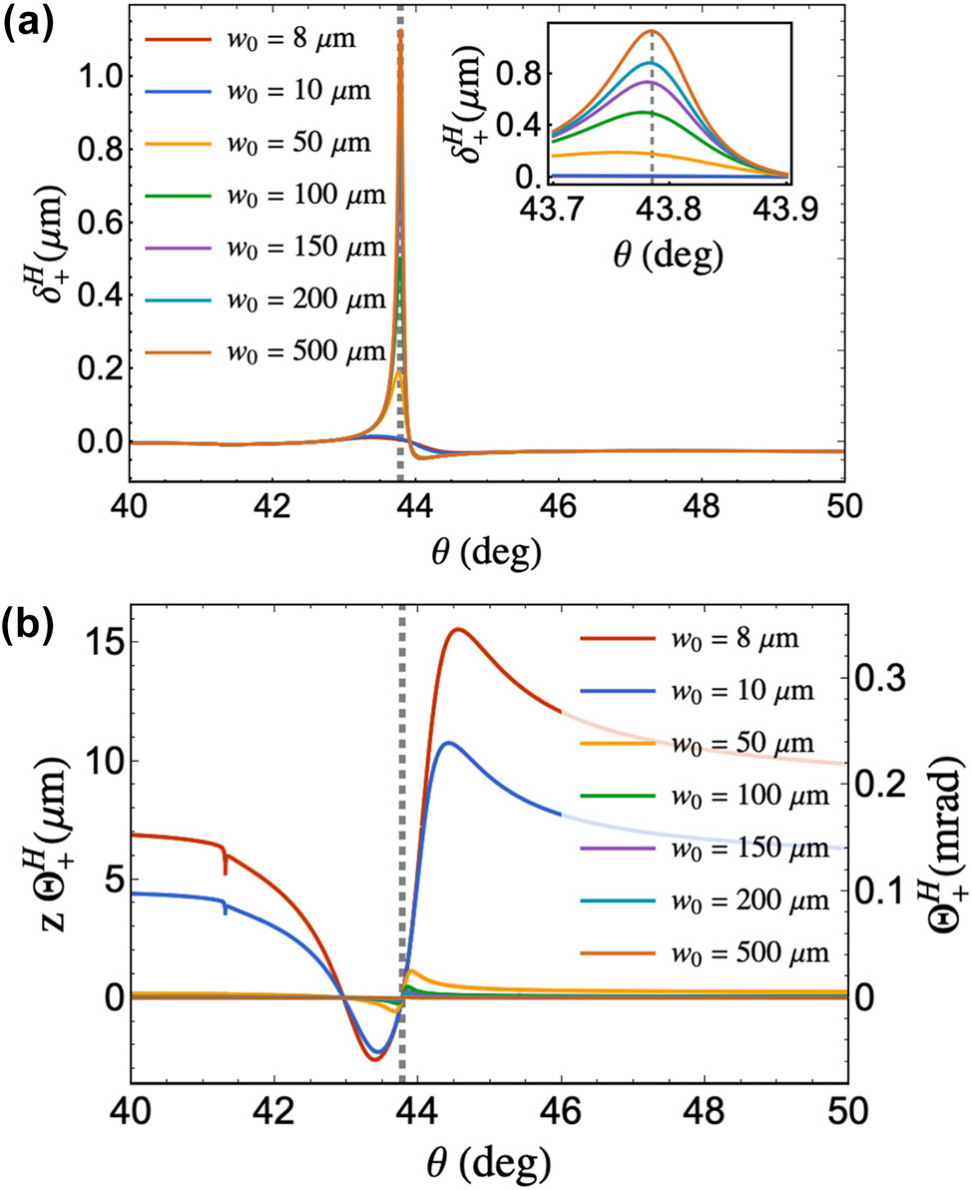
Beam waist dependence of (a) spatial and (b) angular PSHE around the SPR region. The inset in (a) shows a magnified area of the spatial shift to distinctly show the magnitude dependence of the $\delta_{+}^{H}$ with the beam waist. The left *y*-axis in (b) is multiplied by the detector distance *z* = 45,000 μm to show the beam displacement at *z*. The sudden dip in (b) at ∼41.5° corresponds to the critical angle of the structure. The gray dashed lines shown in all plots indicate the calculated SPR angle (*θ*_*SPR*_ = 43.79°) of the structure used.

Figure 2(a) and (b) show the beam waist dependence of the $\delta_{+}^{H}$ and $\Theta_{+}^{H}$ of the LCP component of the reflected beam under *p*-polarized incidence. As shown in Figure 2(a), the calculated spatial transverse shift is maximum at the SPR angle, consistent with calculations from literature for a well-collimated incident beam [21].

**Figure 3:**
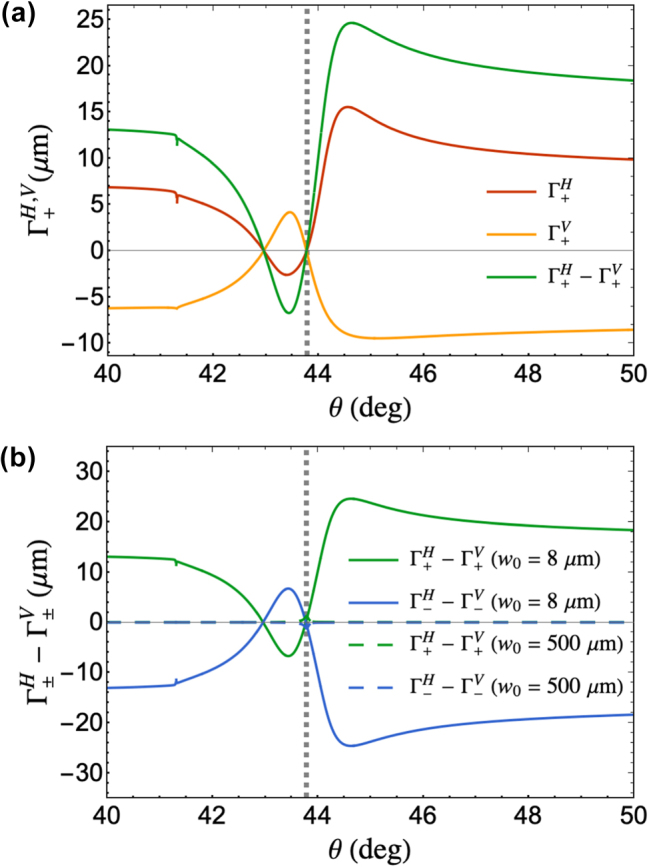
Total transverse shift of the reflected beam. (a) Total shift of the LCP component of the reflected beam under *p*-polarized (red) and *s*-polarized (orange) incidence for *w*_0_ = 8 μm and the detector distance is at 4.5 cm from the location of *w*_0_. Equations used for the vertically polarized incidence are shown in Supplementary Section 2. The difference between the red and orange plots, $\Gamma_{+}^{H}-\Gamma_{+}^{V}$ , is shown in green, which we propose to be used for measurement to obtain higher contrast. (b) Comparison between Γ^*H*^ − Γ^*V*^ for LCP (green) and RCP (blue) components of the reflected beam under plane wave (dashed line) and focused beam (solid line) incidence. The detector distance is at 4.5 cm from the location of *w*_0_. The gray dashed lines in both plots indicate the calculated SPR angle (*θ*_*SPR*_ = 43.79°) of the structure used.

Under *p*-polarized incidence, *r*_*p*_ varies drastically with the incident angle around the SPR region (Figure 1(c)), which means that the wave vector spread becomes significant. Therefore, the ${\left|\partial r_{p}/\partial \theta \right|}^{2}$ term in Eqs. (1) and (2) must be considered for a more accurate calculation of PSHE. Figure 2(a) shows that as the beam waist is decreased, the wave vector spread leads to a decrease in the $\delta_{+}^{H}$ until it becomes negligible for small enough beam waist. Meanwhile, larger $\Theta_{+}^{H}$ at the vicinity of the SPR angle of the reflected beam also arises as shown in Figure 2(b). At the SPR angle, we calculated zero angular shift. The red plot in Figure 3(a) shows $\Gamma_{+}^{H}$ , the total transverse shift of the LCP component of the reflected beam when the incident beam is focused to a beam waist of *w*_0_ = 8 μm and the detector is positioned 4.5 cm from the beam waist.

For typical beam shift measurements at SPR, such as when measuring GH shift [35], [36], [37], beam shift under *s*-polarized incidence serves as the reference position. Here, we propose to modulate between *p* and *s* polarization states during experiment as a contrast enhancement technique. Under *s*-polarized incidence, both $\delta_{+}^{V}$ and $\Theta_{+}^{V}$ depend on *r*_*p*_/*r*_*s*_ ratio (Supplementary Section 2). Because |*r*_*s*_| does not drastically change at the SPR region, $\delta_{+}^{V}$ becomes negligible compared to $\delta_{+}^{H}$ under well-collimated incidence (compare Figures 2(a) and S1(a)). The phase difference between *r*_*p*_ and *r*_*s*_ remains the same at the vicinity of the SPR angle. Thus, $\Theta_{+}^{V}$ is large at the vicinity of the SPR angle albeit opposite in sign as $\Theta_{+}^{H}$ when comparing Figures 2(b) and S1(b). At focused incidence, $\delta_{+}^{H}$ becomes negligible similar to $\delta_{+}^{V}$ making both $\Gamma_{+}^{H}$ and $\Gamma_{+}^{V}$ dominated by $\Theta_{+}^{H}$ and $\Theta_{+}^{V}$ , respectively. As such, for the Θ_+_-dominant PSHE shift under focused incidence, $\Gamma_{+}^{H}$ has almost the same magnitude as $\Gamma_{+}^{V}$ but opposite in sign as shown in Figure 3(a). Therefore, measuring $\Gamma_{+}^{H}-\Gamma_{+}^{V}$ would lead to higher contrast in the measurement. The enhanced values from $\Gamma_{+}^{H}-\Gamma_{+}^{V}$ would then be directly measurable by typical position sensing devices such as quadrant detectors.

The primary finding of our analytical model is summarized in Figure 3(b). We demonstrate the significant enhancement of PSHE induced by SPR. By focusing the incident beam to a small beam waist, we obtained a negligible spatial PSHE and a significantly large angular PSHE. The propagation-dependent angular term dominates the total PSHE when the incident beam is focused to small beam waist and the detector distance is increased. We compare the total PSHE of a focused beam (*w*_0_ = 8 μm) to the total PSHE of a well-collimated beam with *w*_0_ = 500 μm indicated by the plots with dashed line in Figure 3(b). For a well-collimated beam, the spatial PSHE dominates the total PSHE and is approximately similar to the total PSHE under plane wave incidence. As shown in Figure 3(b), we demonstrate more than an order of magnitude increase in the total PSHE via focused beam incidence, which could still be further enhanced by decreasing the beam waist and/or increasing the detector distance. Beam shifts reaching up to tens of microns are directly detectable using a standard position sensing detector. Hence, based on the proposed PSHE enhancement, experimental detection of the shifts could be made by isolating each spin component using a polarimetric-based measurement.

## 3 Experimental methods

Substrates were prepared via electron beam (EB) evaporation on UV/O_3_-cleaned cover slips (*t* = 150 μm) at a base pressure of 4.0 × 10^−4^ Pa. A 2.5 nm of Ti adhesion layer was initially deposited at a rate of 0.5 Å/s followed by 47.5 nm of Au layer at a rate of 1.0 Å/s. The thickness of both deposited layers were calibrated using a quartz microbalance sensor.

**Figure 4:**
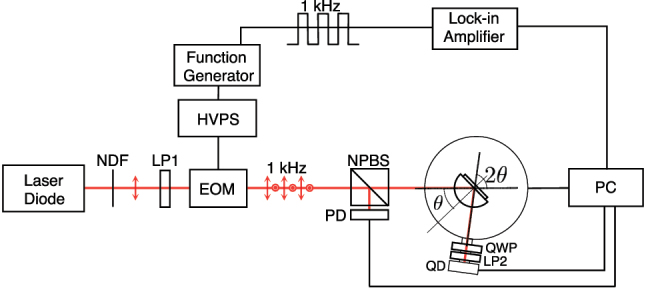
Schematic diagram of the experimental setup. Spin component shift measurements were made by adding the QWP and LP2 before the PSD. The axis of LP2 can be varied by setting the angle *α* with respect to the horizontal. Spin-independent IF shift measurements were performed by removing QWP and LP2. (NDF, neutral density filter; LP, linear polarizer; EOM, electro-optic modulator; NPBS, nonpolarizing beam splitter; PD, photodiode; QWP, quarter waveplate; QD, quadrant detector; HVPS, high voltage power supply).

The schematic diagram of the measurement setup used is shown in Figure 4. The incident source is a linearly polarized laser diode (*λ* = 633 nm). The polarization is switched between the *p* and *s* polarization states using an electro-optic (EO) modulator (Con-optics 350–80) with an extinction ratio of 450:1 and 1 kHz modulation frequency. The beam was separated into the sample beam and the reference beam using a nonpolarizing beam splitter (NPBS). The reference beam was directed toward a photodiode (Thorlabs S120C) while the sample beam was directed toward a *θ* − 2*θ* rotation stage (Sigma-Koki SGSP120YAW) where the sample was mounted. Polarimetric-based measurement to separate the spin components after reflection was done by the combination of the quarter waveplate (QWP) and linear polarizer (LP2). The fast axis of the QWP was placed 45° with respect to the horizontal, while the axis of LP2 was set at an angle *α* with respect to the horizontal. Each spin component was obtained by setting *α* = 0° and *α* = 90°.

Reflectivity measurements were obtained prior to mounting the QWP and LP to find the location of the SPR angle. Reflected beam centroid displacements were obtained using a quadrant detector (Thorlabs PDQ80A, calibration shown in Figure S4) positioned 4.5 cm from *w*_0_ and extracted using a lock-in amplifier (SRS SR830). The EOM modulation frequency was used as a reference, and a 10 ms time constant was used for lock-in detection. The quadrant detector used is capable of measuring beam displacements along the *x* and *y* axis making simultaneous measurements of both GH and IF shifts possible.

The substrates were mounted on the flat side of a BK7 hemispherical prism (*D* = 25 mm) using an index-matching oil (*n*_oil_ = 1.518). To accommodate the thickness of the glass substrate, the flat side of the prism was polished by 150 μm. The incident beam was introduced on the curved side of the prism, which focuses the incident beam to a small beam waist.

## 4 Results and discussion

**Figure 5:**
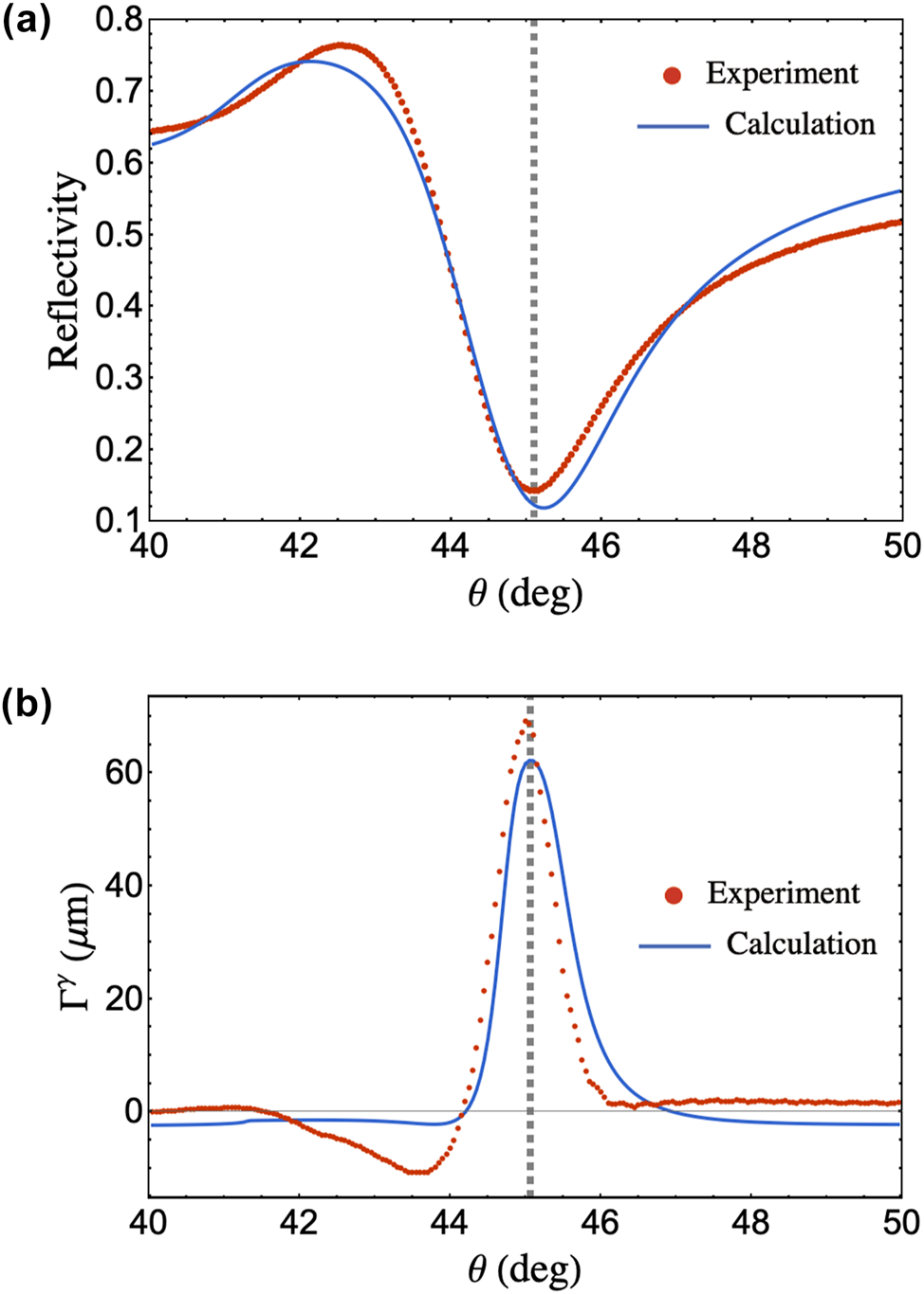
Experimental and analytical values of the (a) reflectivity and (b) IF shift of the reflected beam centroid around the SPR region. The gray dashed line corresponds to the SPR angle located at 45.1°.

Experimental measurements made around the SPR region without spin isolation are shown in Figure 5. Our measurements showed a dip in the reflectivity with the SPR angle located at 45.1°, indicated by the gray dashed line in the plot shown in Figure 5(a). Difference in the location of the SPR angle obtained from analytical calculations could be accounted for by the addition of the Ti adhesion layer, the actual deposited film thickness of both the Ti and Au layer, and the deposition-dependence of the complex permittivity of the deposited layers. By performing a nonlinear least square curve fit of the reflectivity and GH shift similar to the procedure performed in Ref. [37], we obtained the experimental values of the complex refractive index of the effective metal layer at *n*_eff_ = 0.158 + 2.855*i* and the minimum beam waist of the focused incident beam, *w*_0_ = 7.995 μm (see Supplementary Section 3). The extracted *w*_0_ compare well with matrix-based beam propagation calculation of beam waist at *w*_0_ = 8.6 μm. Reflectivity plot based on these parameters is shown in Figure 5(a).

Figure 5(b) shows the IF shift, Γ^*γ*^ measured without spin isolation and obtained simultaneously with reflectivity in Figure 5(a) and GH shift in Figure S2. Because the LCP and RCP components of the reflected beam will be shifted in equal magnitudes but in opposite directions as shown in Figure 3(b), Γ^*γ*^ obtained without spin isolation is expected to be zero.

The sharp peak in the SPR angle observed in Figure 5(b) can be attributed to the strong polarization dependence of the IF shift, where even slight polarization deviation during measurement could result in a large IF shift. Analytical expressions of IF shifts for arbitrarily polarized beams in Ref. [27] demonstrate that reflected beams experience an IF shift when the incident polarization is no longer pure *p* and pure *s*. Here, *γ* indicates the polarization angle where *γ* = 0 corresponds to a *p* polarization state and *γ* = 90° corresponds to an *s* polarization state. Based on their work, we showed in Figure S3(a) that a sharp Γ^*γ*^ would occur at SPR for small polarization deviations when the incident beam is focused to a small beam waist.

Analytically, Γ^*γ*^ = 0 at the SPR region when a pure *p* and pure *s* polarized incident source is used (*γ* = 0 and *γ* = 90° in Supplementary Figure S4, respectively). However, our experimental measurements demonstrate a significant peak at the SPR angle with Γ^*γ*^ = 68.8 μm. As such, this peak measured at the SPR angle could primarily be induced by the practically limited polarization purity in the measurements. We extracted this polarization deviation by performing a curve fit in the measured Γ^*γ*^ using the experimental parameters obtained from reflectivity and GH shift curve fit. We obtained *γ*_*p*_ = −10.7° and *γ*_*s*_ = 26.7° with respect to the *p*-polarized and *s*-polarized directions, respectively, taking into account the polarization switching during measurement.

Polarization deviations could be attributed to the inherent extinction of the EOM and the roughness of the film used. In our work, the relatively high extinction ratio of the EOM and the low RMS roughness of the Au film at 430 pm (Atomic Force Microscopy (AFM) image shown in Supplementary Figure S3) indicates that these factors are negligible contributors to polarization deviation, which supports the robustness of our method as a surface sensing technique. The calculated Γ^*γ*^ based on the extracted *γ*_*p*_ and *γ*_*s*_ in Figure 5(b) shows that polarization deviations induced the sharp peak at the SPR angle. However, considering the large values extracted and the goodness of fit, other factors could have also induced an IF shift that are otherwise not directly extracted from the analytical expression of Γ^*γ*^ in Ref. [27].

**Figure 6:**
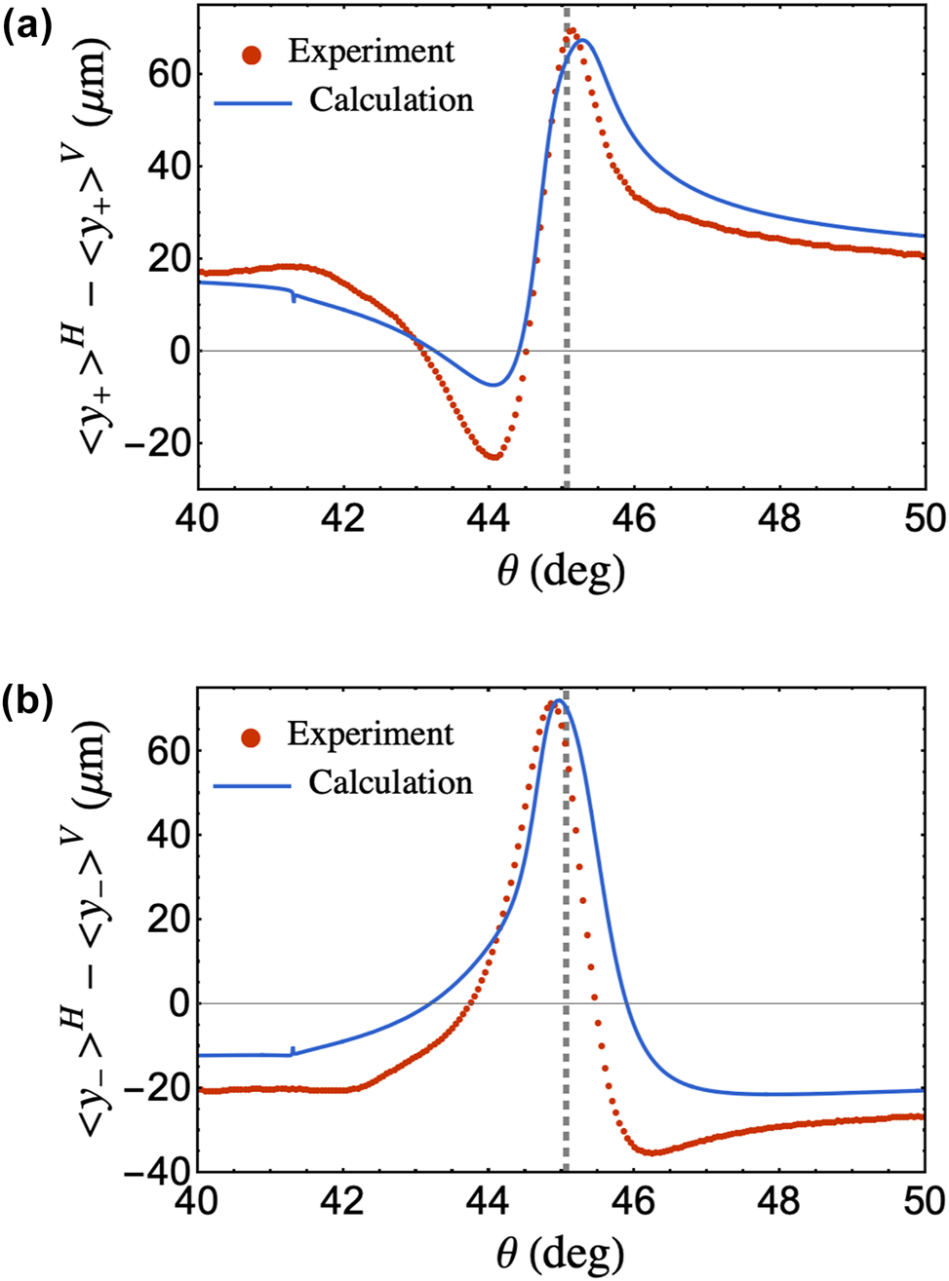
Experimental and analytical values of the transverse displacement of the (a) LCP and (b) RCP spin components of the reflected beam centroid around the SPR region. Values used for the analytical calculations on both plots are the experimental parameters obtained via nonlinear least square curve fit of reflectivity, GH shift and IF shift measurements. The gray dashed lines correspond to the SPR angle located at 45.1°.

Spin component measurements of the reflected beam centroid, $\left\langle y_{\pm }\right\rangle$ , are shown in Figure 6 with the gray dashed line indicating the location of the SPR angle. Figure 6(a) is obtained when LP2 was set at *α* = 90° corresponds to the LCP component of the reflected beam. Meanwhile Figure 6(b), which is obtained when LP2 was set at *α* = 0°, corresponds to the RCP component of the reflected beam.

**Figure 7:**
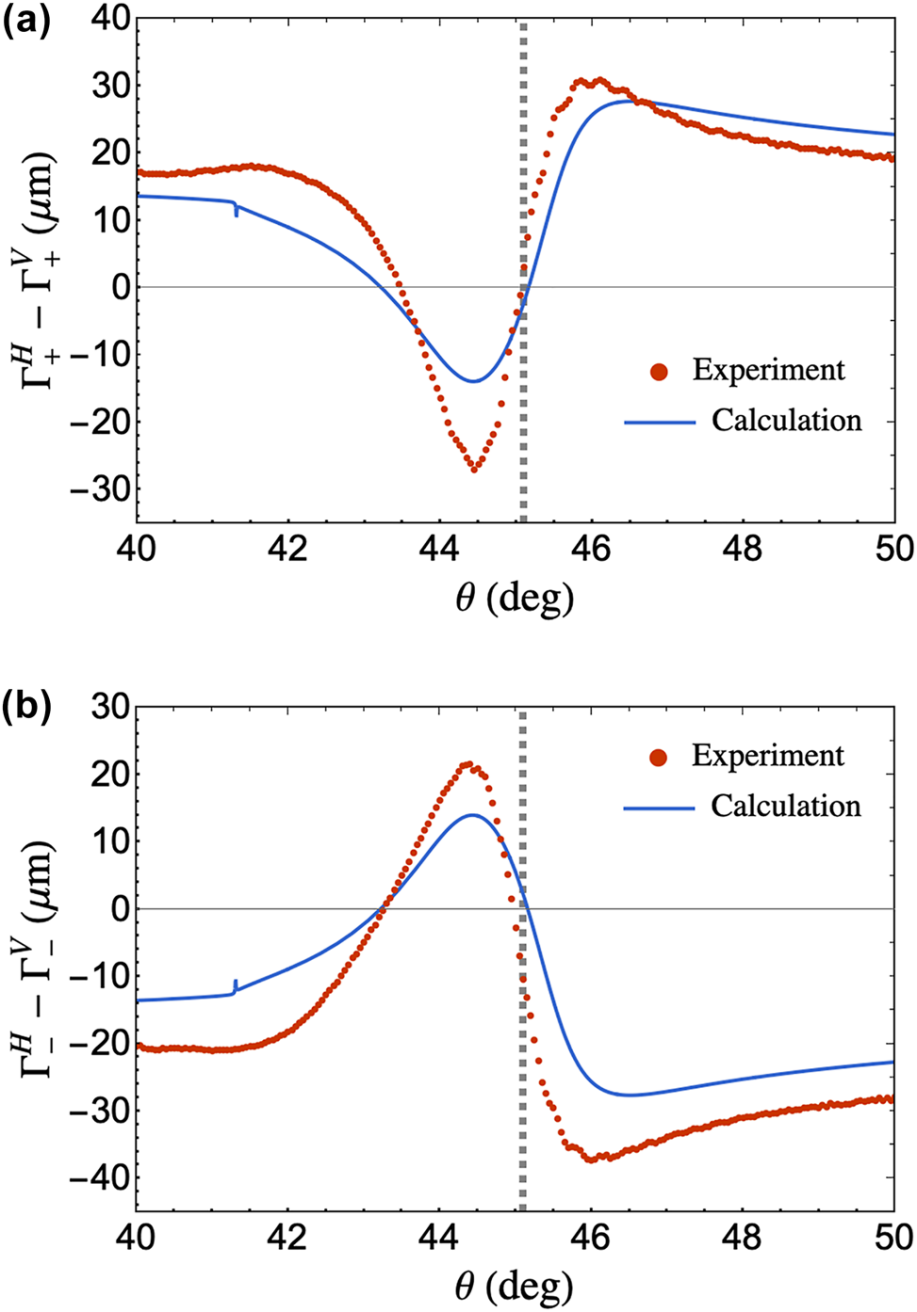
Experimental and analytical values of the (a) LCP and (b) RCP PSHE spin component shift of the reflected beam obtained by subtracting the corrective IF shift, Γ^*γ*^, from the measured spin component reflected beam centroid, $\left\langle y_{\pm }\right\rangle$ , along the transverse direction. Values used for the calculations on both plots are the experimental parameters obtained via nonlinear least square curve fit of reflectivity, GH shift and IF shift measurements. The gray dashed lines correspond to the SPR angle located at 45.1°.

As a transverse shift in addition to the PSHE spin splitting, Γ^*γ*^ serves as a corrective shift owing to the polarization deviations. Essentially, Γ^*γ*^ induces an overall transverse shift of the reflected beam, which translates to the asymmetry in $\left\langle y_{\pm }\right\rangle$ shown in Figure 6, which corresponds to the shift of the reflected beam centroid of the left and right spin components with respect to the plane of incidence. As such, $\left\langle y_{\pm }\right\rangle$ becomes

(3) $${\left\langle y_{\pm }\right\rangle }^{H}-{\left\langle y_{\pm }\right\rangle }^{V}=\Gamma_{\pm }^{H}-\Gamma_{\pm }^{V}+\Gamma^{\gamma },$$

where Γ_±_ is the symmetric PSHE spin component shift. Based on Eq. (3), the symmetric Γ_±_ undergoes an additional shift Γ^*γ*^ leading to the asymmetric $\left\langle y_{\pm }\right\rangle$ shown in Figure 6 in agreement with other works [30], [31], [38]. The analytical calculation of the asymmetric $\left\langle y_{\pm }\right\rangle$ compares well with the experimental measurements shown in Figure 6. To obtain the PSHE shift of the left and right spin components, we subtract Γ^*γ*^ to $\left\langle y_{\pm }\right\rangle$ . This implies that Γ_±_ is the transverse shift of the left and right spin components with respect to Γ^*γ*^. After subtraction, the experimentally measured spin components is shown in Figure 7, which is in agreement to the calculated values based on experimental parameters.

Our results demonstrate an Θ_±_-dominated PSHE with a crossing point at Γ_±_ = 0 at the SPR angle. We measured a maximum displacement magnitude of ∼37μ m within the vicinity of the SPR angle. This magnitude is more than 30 times greater than the maximum displacement magnitude should a well-collimated beam is considered for the same material structure. The Θ_±_-dominated PSHE measurement is induced by focusing the incident beam to a small beam waist, which also made the contribution of *δ*_±_ negligible.

As shown in Figures 6 and 7, experimentally measured spin component shifts generally agree with the analytical trend, although certain regions exhibit shifts larger than calculated values. While our work has accounted for the effect of polarization deviation inducing a corrective IF shift, other factors such as the integration effect of the detector could also induce an overestimation in the measured shift. The QD that we used is calibrated for a Gaussian beam. Especially for larger incident angles, the reflected beams would be distorted and have asymmetries leading to measurement displacements beyond analytical predictions. Nonetheless, the overall agreement still supports the validity of the method we presented.

Currently, there is no reported literature that measures PSHE of Au film under surface plasmon excitation in the Kretschmann configuration using weak measurement. However, existing studies employing weak measurements to measure PSHE in nanometal films [10] and the SPR of Au film with graphene [22] have reported shifts an order of magnitude larger than in our current work. While weak measurements can indeed produce much larger shifts, we emphasize that the angular PSHE enhancement in our approach provides controllability via beam propagation parameters, allowing tuning of total spin-dependent shifts. Thus, even larger spin component shifts than currently demonstrated are achievable limited only by the detector size. Moreover, the near-orthogonal postselection used in weak measurements significantly reduces signal intensity. In contrast, our use of polarimetric measurement to isolate the spin components provides higher signal intensity, which would enable more robust signal detection. Therefore, our approach offers a practical and direct route to observe PSHE, making it advantageous for applications where stronger signal is still necessary such as when coupling PSHE to near-field interactions and plasmonic sensing.

**Figure 8:**
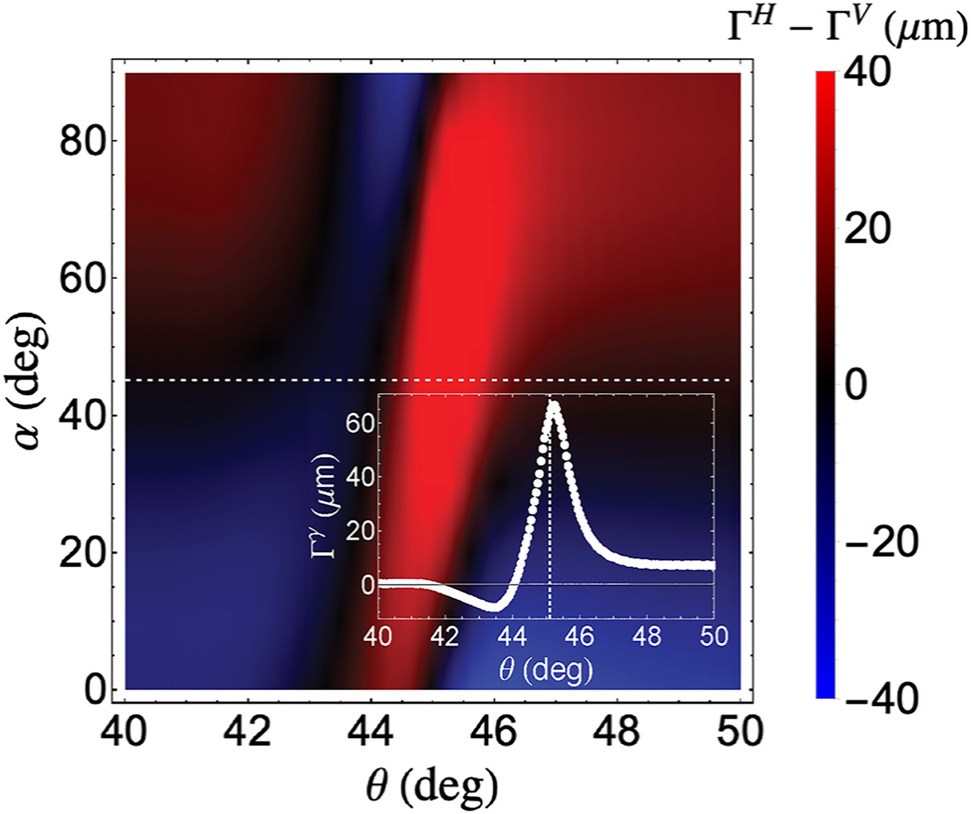
PSHE spin component shift at varying LP2 orientation after IF shift correction. Inset shows the IF-corrected shift when the LP2 orientation is at *α* = 45°, which resembles the Γ^*γ*^ plot shown in Figure 5(b).

Aside from measuring the spin component displacement, the polarimetric scheme used in our system allows for the measurement of the displacement measurement of other polarization directions, *α*, in the reflected beam as shown in Figure 8. Outside the SPR region, the Γ^*H*^ − Γ^*V*^ changes linearly as *α* is increased indicating that the RCP component of the reflected beam decreases and is eventually transformed to LCP component. At the SPR region, however, a large displacement with a peak at the SPR angle was observed when *α* = 45° as shown in the inset of Figure 8. At this LP2 orientation, both RCP and LCP components of the reflected beam are detected, similar to the polarization of the beam before passing through the QWP and LP2. As such, Γ^*H*^ − Γ^*V*^ observed at *α* = 45° has similar trend as that of Γ^*γ*^.

The significance of our result becomes apparent when we consider the potential applications of PSHE measurement. Because our measurements are made at SPR, an immediate application is for high sensitivity SPR-based sensors where PSHE displacements are measured when there is refractive index change in the sensing layer [21], [22], [23], [24], [25]. The enhanced and direct measurement of spin component shifts can also enable precision metrology for nanoscale displacement detection. Furthermore, the ability to isolate and control spin component displacements without weak measurements can be extended to advanced optical imaging techniques, such as high-contrast microscopy where the spin-dependent information can improve the contrast and resolution [39], [40]. The experimental demonstration presented here improves the feasibility of these applications by providing a direct approach that eliminates the need for complex pre- and postselection, and signal amplification schemes necessary in weak measurements. Our polarimetric-based setup, combined with angular PSHE enhancement via incident beam focusing, thus paves the way for practical and robust spin-controlled functionalities.

## 5 Conclusions

We demonstrated a significantly large PSHE spin component shift upon excitation of surface plasmons on a Au film. The total PSHE shift is dominated by the angular shift induced by focusing the incident beam to a small beam waist. Polarimetry-based optical setup was used to directly measure the Θ_±_-dominated PSHE spin component shift. Our results showed that the total spin component shift of the reflected beam centroid is a combination of the PSHE shift and an additional IF shift. The IF shift arises from the slight deviation in the polarization direction of the incident beam, which becomes more prominent for focused incident beams. We showed that the magnitude of the Θ_±_-dominated PSHE measurements in our work is significantly larger than calculations in literature for a well-collimated beam, circumventing the need for complex measurement schemes.

## Supplementary Material

Supplementary Material Details
