# Supplementary material for: Enhancing angular photonic spin Hall effect at surface plasmon resonance
Source: Nanophotonics. 2025 Sep 8;14(19):3115–24. doi: 10.1515/nanoph-2025-0206 (PMC12455412; doi:10.1515/nanoph-2025-0206)
Supplement: Supplementary file 1 — Supplementary Material Details [file j_nanoph-2025-0206_suppl_001.pdf]

Enhancing angular photonic spin Hall effect at surface plasmon resonance: supplemental document

Cherrie May Olaya[1], Norihiko Hayazawa[1,2], Maria Herminia Balgos[3], Takuo Tanaka[1]

[1] Metaphotonics Research Team, RIKEN Center for Advanced Photonics, Japan
[2] Surface and Interface Science Laboratory, RIKEN Cluster for Pioneering Research, Japan
[3] Center for Quantum Conversion Research, Institute for Basic Science, Gwangju, Republic of Korea

1 Derivation of spatial and angular PSHE

Spin-dependent splitting calculations were performed by considering the angular spectrum of the electric fields. Here we considered the Cartesian coordinate (x, y, z) with the z axis normal to the interface at $z = 0$. We also considered the incident coordinate (x_i, y_i, z_i) and the reflection coordinate (x_r, y_r, z_r) for the incident and the reflected fields, respectively. Fig. 1a in the main text shows the schematic representation of the coordinate system used in our calculation.

For an arbitrarily polarized incident Gaussian beam, the electric field is given by [1]

$$E_i = \mathbf{E}_i^H \hat{\mathbf{e}}_{ix} + \mathbf{E}_i^V \hat{\mathbf{e}}_{iy} \propto (f_p \hat{\mathbf{e}}_{ix} + f_s \hat{\mathbf{e}}_{iy}) \exp\left[-\frac{k_0}{2}\frac{x_i^2 + y_i^2}{L + iz_i}\right] \tag{1}$$

where $\mathbf{E}_i^H$ and $\mathbf{E}_i^V$ are the electric fields along the x_i and y_i direction, respectively, H and V denotes the horizontal and vertical polarization with respect to the plane of incidence, $L = k_0 w_0^2/2$ is the Rayleigh length, and k_0 is the wavenumber in free space. The polarization of the incident beam is determined by $\hat{\mathbf{f}} = (f_p \hat{\mathbf{e}}_{ix} + f_s \hat{\mathbf{e}}_{iy})/(|f_p|^2 + |f_s|^2)^{1/2}$ where p and s refer to the p and s plane waves in the Fresnel formulas. H occurs when $f_p = 1$ and $f_s = 0$ while V occurs when $f_p = 0$ and $f_s = 1$.

The reflected electric field is obtained by employing Fourier transformations [1–3]

$$\mathbf{E}_r = \int dk_{rx} dk_{ry} \tilde{\mathbf{E}}_r \exp\left[i(k_{rx}x_r + k_{ry}y_r + k_{rz}z_r)\right] \tag{2}$$

where $k_{rz} = \sqrt{k_0^2 - (k_{rx}^2 + k_{ry}^2)}$ and the angular spectrum of the reflected field $\tilde{\mathbf{E}}_r$ is obtained from the angular spectrum of the incident beam $\tilde{\mathbf{E}}_i$ by satisfying the boundary conditions of the electric field

$$\tilde{\mathbf{E}}_r = \begin{bmatrix} \tilde{\mathbf{E}}_r^H \\ \tilde{\mathbf{E}}_r^V \end{bmatrix} = \begin{bmatrix} r_p & \frac{k_{ry}\cot\theta_i (r_p + r_s)}{k_0} \\ -\frac{k_{ry}\cot\theta_i (r_p + r_s)}{k_0} & r_s \end{bmatrix} \begin{bmatrix} \tilde{\mathbf{E}}_i^H \\ \tilde{\mathbf{E}}_i^V \end{bmatrix} \tag{3}$$

where r_A with $A \in \{p, s\}$ is the Fresnel reflection coefficient for the p and s polarization states.

Because of the distributed plane wave components of the incident beam, the Fresnel reflection coefficient would only correspond to the central wave vector. To account for the non-central wave vector components, we take the Taylor series expansion at $k_{ix} = 0$ of r_p and r_s up to the first order approximation given by [4–6]

$$r_A(k_{ix}) = r_A(k_{ix} = 0) + k_{ix}\left[\frac{\partial r_A(k_{ix})}{\partial k_{ix}}\right]_{k_{ix}=0}. \tag{4}$$

Typical calculations for PSHE considers approximations up to the zeroth order only. While this is generally sufficient, Refs. [4–6] have shown that wave vector spread affects the values around the Brewster angle when considering an air-dielectric interface. In this work, we show that this effect extends to materials that exhibit sudden changes in the Fresnel coefficients.

We can write the reflected filed in spin basis set to decompose to the LCP and RCP components. In the spin basis set, $|\sigma\rangle = (|H\rangle + i\sigma|V\rangle)/\sqrt{2}$ where $\sigma = \pm 1$ corresponds to the left and right spin states or the LCP and RCP components, respectively. Combining Eqs. 1 - 4, the reflected electric field can be written as [1,5]

$$\begin{aligned}\mathbf{E}_{r\sigma} \propto \exp\left[-\frac{k_r}{2}\frac{x_r^2 + y_r^2}{L + iz_r}\right]\Bigg[f_p r_p - i\sigma f_s r_s - \left(if_p\frac{\partial r_p}{\partial \theta} + \sigma f_s \frac{\partial r_s}{\partial \theta}\right)\frac{x_r}{L + iz_r} \\ + (if_s - \sigma f_p)(r_p + r_s)\cot\theta \frac{y_r}{L + iz_r}\Bigg] \times |\sigma\rangle.\end{aligned} \tag{5}$$

The PSHE manifests itself as a transverse shift of each spin component which is determined from the location of the reflected beam centroid of each spin component. For any given plane in the reflected field z_r, the transverse displacements can be calculated by

$$\Gamma_{\pm} = \frac{\iint y_r |\mathbf{E}_{r\pm}|^2 dx_r dy_r}{\iint |\mathbf{E}_{r\pm}|^2 dx_r dy_r} \tag{6}$$

which, as visualized in Fig. 1b in the main text, can be separated into non-propagating and propagating terms [1, 7] such that

$$\Gamma_{\pm} = \delta_{\pm} + z_r \Theta_{\pm} \tag{7}$$

where

$$\delta_{\pm} = \mp \frac{k_0 w_0^2 \,\mathrm{Re}\left[\left(f_p r_p \mp i f_s r_s\right)\left(f_p \pm i f_s\right)\left(r_p + r_s\right)^*\right] \cot\theta}{k_0 w_0^2 \left|f_p r_p \mp i f_s r_s\right|^2 + \left|f_p \frac{\partial r_p}{\partial \theta} \mp i f_s \frac{\partial r_s}{\partial \theta}\right|^2 + \left|\left(r_p + r_s\right)\cot\theta\right|^2} \tag{8}$$

$$\Theta_{\pm} = \pm \frac{2 \,\mathrm{Im}\left[\left(f_p r_p \mp i f_s r_s\right)\left(f_p \pm i f_s\right)\left(r_p + r_s\right)^*\right] \cot\theta}{k_0 w_0^2 \left|f_p r_p \mp i f_s r_s\right|^2 + \left|f_p \frac{\partial r_p}{\partial \theta} \mp i f_s \frac{\partial r_s}{\partial \theta}\right|^2 + \left|\left(r_p + r_s\right)\cot\theta\right|^2} \tag{9}$$

where $\delta_{\pm}$ and $\Theta_{\pm}$ correspond to the spatial and angular components of PSHE, respectively, and * is the complex conjugate of the term. Here, we assume that $\Theta_{\pm} \ll 1$ so that $\tan^{-1}(\Theta_{\pm}) \approx \Theta_{\pm}$. Typical calculations for PSHE considers up to Eq. 8 only which is obtained by solving Eq. 6 at $z = 0$ and making the propagating $\Theta_{\pm}$ term negligible. This approximation is valid if the incident beam is well-collimated. Because our work focuses on the spin component shift under focused beam incidence, the beam waist-dependent propagating $\Theta_{\pm}$ term is no longer negligible.

Under p-polarized incidence, Eqs. 8 and 9, respectively, simplify to

$$\delta_{\pm}^{H} = \mp \frac{k_0 w_0^2 \left|r_p\right|^2 \mathrm{Re}\left[1 + \frac{r_s}{r_p}\right] \cot\theta}{k_0 w_0^2 \left|r_p\right|^2 + \left|\frac{\partial r_p}{\partial \theta}\right|^2 + \left|\left(r_p + r_s\right)\cot\theta\right|^2} \tag{10}$$

$$\Theta_{\pm}^{H} = \pm \frac{2 \left|r_p\right|^2 \mathrm{Im}\left[1 + \frac{r_s}{r_p}\right] \cot\theta}{k_0 w_0^2 \left|r_p\right|^2 + \left|\frac{\partial r_p}{\partial \theta}\right|^2 + \left|\left(r_p + r_s\right)\cot\theta\right|^2}. \tag{11}$$

which are the main equations used in the main text.

2 Beam waist dependence of spatial and angular PSHE under vertical polarization

Calculation of PSHE under vertically-polarized (s-polarized) incidence was made using Eqs. 10-11 in the main text upon switching the p and s subscripts of the Fresnel coefficients given by

$$\delta_{\pm}^{V} = \mp \frac{k_0 w_0^2 \left|r_s\right|^2 \mathrm{Re}\left[1 + \frac{r_p}{r_s}\right] \cot\theta}{k_0 w_0^2 \left|r_s\right|^2 + \left|\frac{\partial r_s}{\partial \theta}\right|^2 + \left|(r_s + r_p)\cot\theta\right|^2} \tag{12}$$

$$\Theta_{\pm}^{V} = \pm \frac{2\left|r_s\right|^2 \mathrm{Im}\left[1 + \frac{r_p}{r_s}\right] \cot\theta}{k_0 w_0^2 \left|r_s\right|^2 + \left|\frac{\partial r_s}{\partial \theta}\right|^2 + \left|(r_s + r_p)\cot\theta\right|^2}. \tag{13}$$

Fig. S1 shows the beam waist dependence of both the spatial and angular terms. δ_{+}^{V} plots are mainly dependent on the $\mathrm{Re}\,(1 + r_p/r_s)$. Because there is no sudden change in r_s value, the second and third terms of the denominator can be considered negligible. Hence, the equation would still degenerate to the transverse shift derived in Ref. [8] which does not show any dependence to w_0 as shown in Fig. S1a. The angular term, however, in Fig. S1b is still dependent on w_0. Owing to its propagation dependence, smaller w_0 leads to larger Θ_{+}^{H} as shown in Fig. S1b.

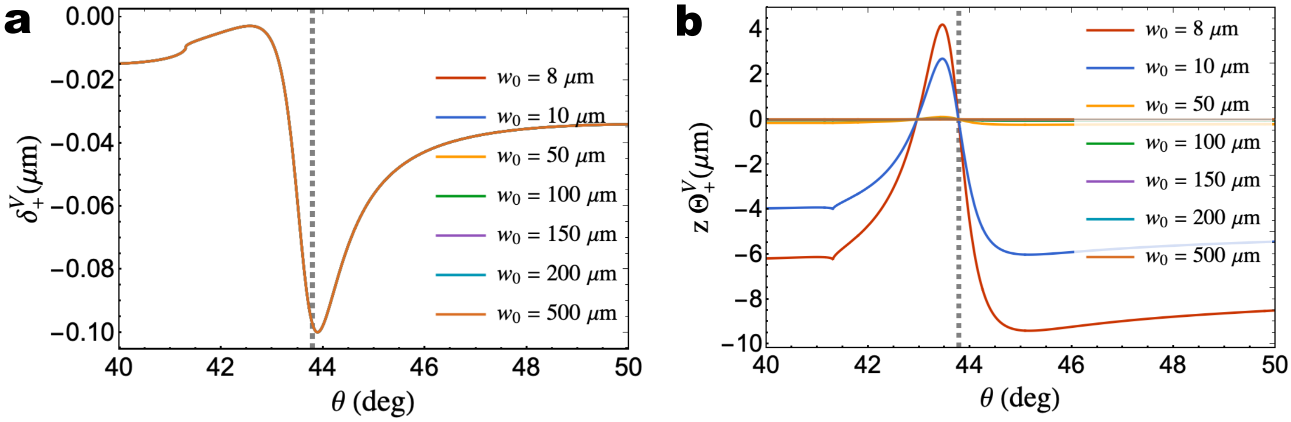

Figure S1: (a) Spatial PSHE and (b) angular PSHE around the SPR region under vertical polarization at varying beam waist values. No beam waist dependence occurs in (a) and the plots completely overlap each other. For both (a) and (b), the detector distance is set at 4.5 cm from the location of w_0.

3 Reflectivity and GH shift data curve fit

Experimental measurements of reflectivity and GH shift measurements as shown in S2 were obtained as described in the main text which were both performed simultaneously as the IF shift measurements. We determined the complex refractive index of the effective metallic layer of $n_{eff} = 0.158 + 2.855i$ and the beam waist of the beam incident on the substrate of $w_1 = 11.62$ μm using the nonlinear least square curve fit using the Levendberg-Marqaudt algorithm of the reflectivity values. The experimental plot and the fitted curve are shown in Fig. S2a

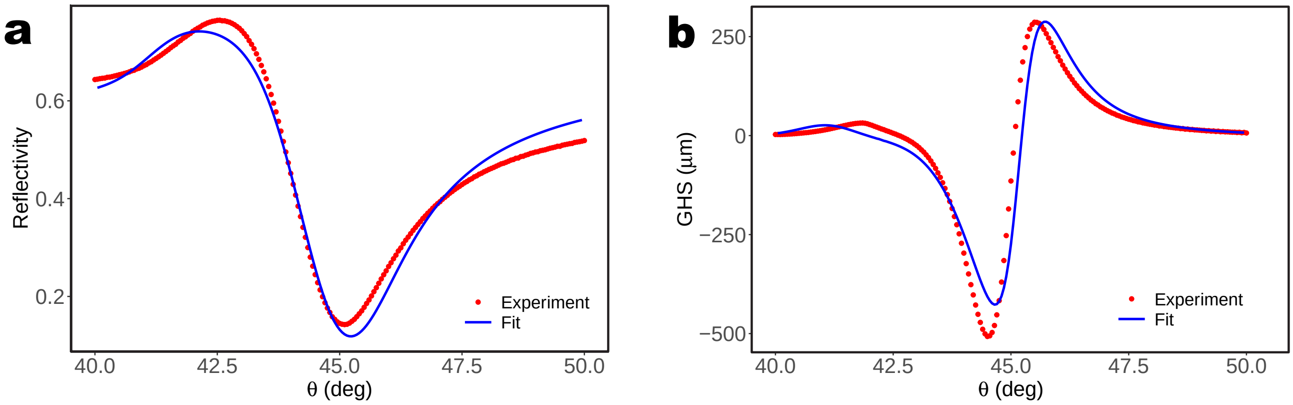

Figure S2: Experimental data (red dot) and fitting curve of the (a) reflectivity and (b) GH shift measurement.

We used the parameters obtained from the reflectivity fit as constant parameters to perform another nonlinear least square curve fit of GH shift measurement to extract the minimum beam waist of the reflected beam after passing through the prism-air interface. The extracted value of $w_0 = 7.995$ μm compare well with matrix based calculation of beam waist at 8.6 μm.

4 AFM Image of Evaporated Au Film Sample

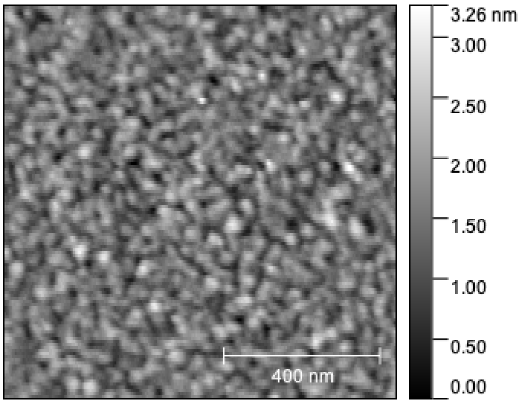

Figure S3: AFM image of evaporated Au film sample used during the measurement. Image analysis showed RMS roughness of 430 pm. The surface roughness of the film could also induce cross-polarization during measurement which could add to polarization mixing during measurement.

5 IF shift correction

According to Aiello, et al. [9], Imbert-Fedorov (IF) shift for an arbitrarily polarized beam is given by

$$\langle Y_r \rangle = -\frac{a_p a_s \cot\theta}{R_p^2 a_p^2 + R_s^2 a_s^2} \left\{ \left[\left(R_p^2 + R_s^2 \right) \sin\eta + 2 R_p R_s \sin\left(\eta - \phi_p + \phi_s \right) \right] - \frac{Z_r}{\Lambda} \left(R_p^2 - R_s^2 \right) \cos\eta \right\} \tag{14}$$

where $r_A = R_A \exp(i\phi_A)$ is the Fresnel coefficient, $A \in \{s, p\}$. The polarization direction $\hat{\mathbf{f}}$ is defined in the main text where the polarization components are $f_p = a_p$, $f_s = a_s \exp(i\eta)$, and η is the phase difference. Z_r is the propagation distance of the beam and $\Lambda = kL$ where $L = kw_0^2/2$ is the Rayleigh range and k is the wavenumber of the incident beam in free space.

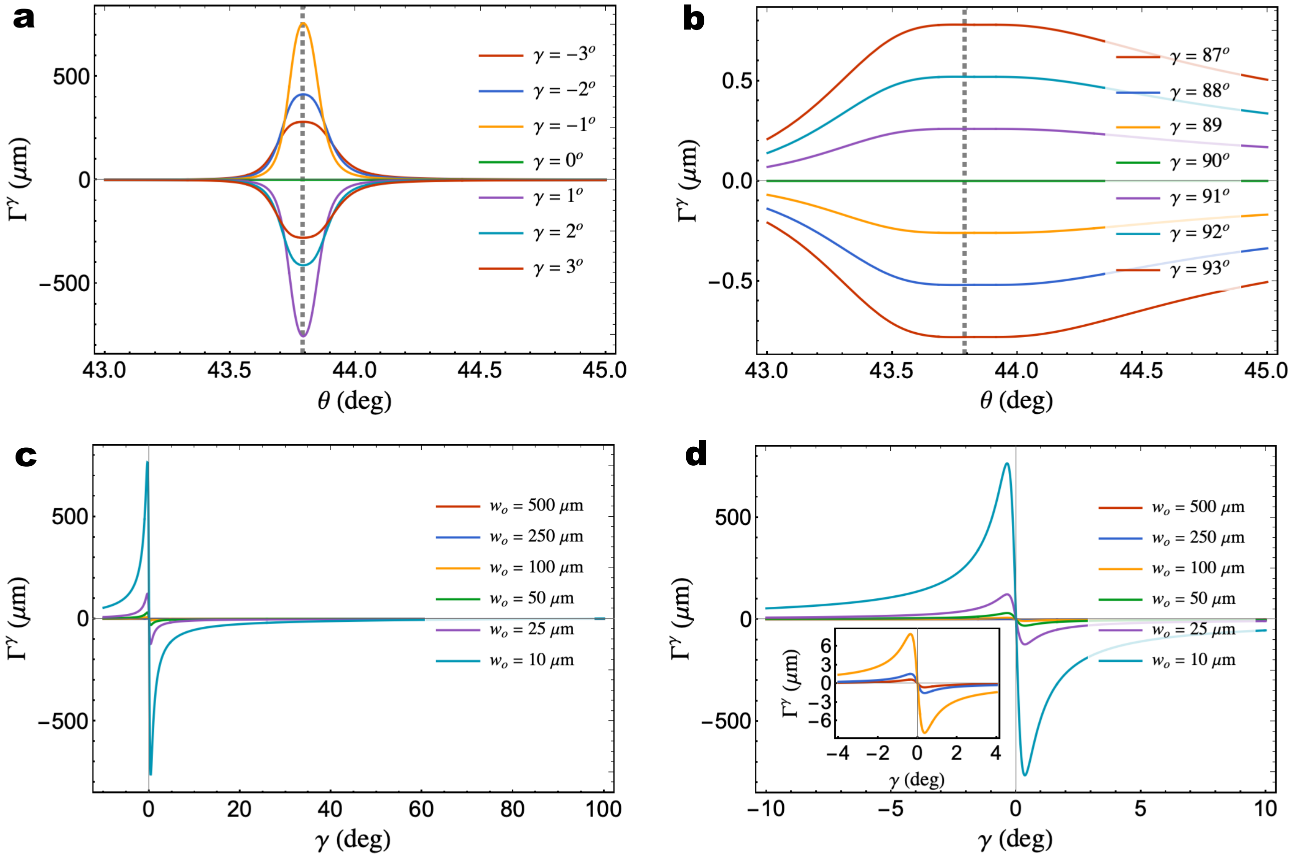

Figure S4: IF shift for $w_0 = 8\mu$m when there is a slight deviation in the polarization direction with respect to (a) a p-polarized and (b) a s-polarized illumination for a configuration similar as in the main text. The grey dashed line corresponds to the location of the SPR angle. The detector distance is set at 4.5 cm from the location of w_0. (c) Beam waist dependence of IF shift at the SPR angle at increasing values of angle of polarization. Sharp Γ^γ was observed only at near $\gamma = 0$ corresponding to a pure p polarized incidence. (d) Beam waist dependence of IF shift at the SPR angle for a smaller polarization angle range to more visibly see the sudden increase and decrease in IF shift values under focused incidence. The inset is a zoomed in version for larger values of beam waist showing zero to negligible IF shift at quasi-collimated incidence.

For a linearly polarized beam with a slight change in polarization direction, $\eta = 0$, $a_p = \cos\gamma_i$ and $a_s = \sin\gamma_i$ where γ_i is the angle of polarization direction. Fig. S4a shows the IF shift, Γ^γ at varying polarization deviation of the incident angle with respect to a p-polarized source. At $\gamma = 0$, $\Gamma^\gamma = 0$ which is expected for IF shifts since IF shift does not have an eigenmode at p and s polarization. A very sharp peak is observed at the SPR angle upon addition of a small polarization deviation which decreases as the polarization deviation is increased. This is in contrast to when a slight polarization deviation with respect to the pure s polarization occurs as shown in Fig. S4b. While there is also a peak around the SPR region when there is a slight polarization change, this peak is orders of magnitude smaller as compared with the peak near the p polarized incidence. As such the appearance of the sharp peak is indeed induced by the excitation of surface plasmons. Fig. S4c-d shows the beam waist dependence of the IF shift at the SPR angle as the amount of polarization is increased. A sudden increase in Γ^γ is observed upon addition of polarization deviation which reached a maximum value at $\sim 0.5°$. Then, Γ^γ slowly decreases as γ is further increased. These values become zero as $\gamma = 90°$ since no IF shift is expected for a pure s polarized incidence.

The focused incidence also takes advantage of the propagation dependence of Γ^{γ}. Fig. S4a-b is thus significantly dependent on the $-\frac{Z_r}{\Lambda}\left(R_p^2 - R_s^2\right)\cos\eta$ term in Eq. 14. Hence, as shown in Fig. S4b, the peak at the SPR angle is much significant for smaller w_0 values and almost negligible when a well-collimated is assumed (i.e $w_0 = 500$ μm).

6 Reproducibility Confirmation

Fig. S5 shows a IF shift and PSHE measurements performed to confirm the resproducibility of the measurements shown in the manuscript. The values of the measurements are similar to the measurements shown in the main text albeit with some measurement artifact.

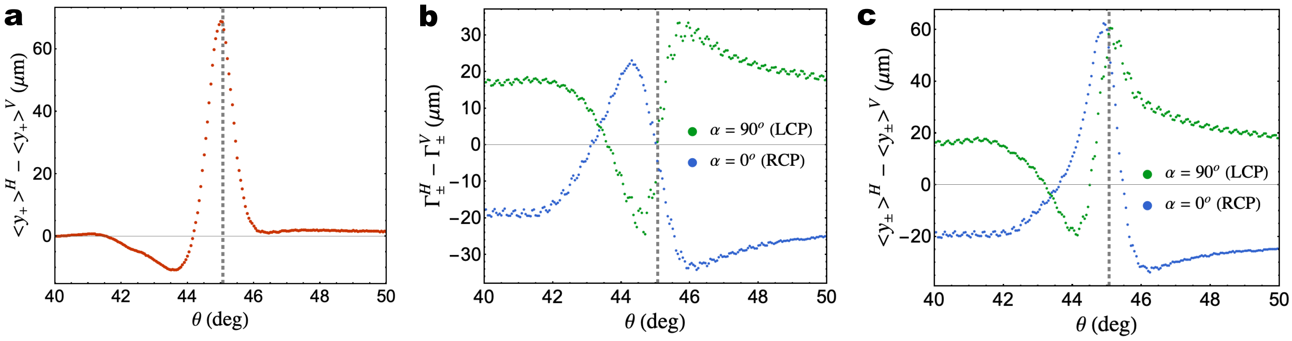

Figure S5: (a) IF shift measurement measured around the SPR region. PSHE measurements (b) with and (c) without the IF shift for both the RCP and LCP spin components. The grey dashed lines correspond to the SPR angle located at 45.1°.

7 Quadrant detector calibration

Quadrant detectors (QD) are composed of four photodiodes arranged in a quadrant separated by a gap. It works by profiling the intensity distribution within each segment requiring that each segment must have a portion of the beam. Because the current or voltage outputs of QD become nonlinear as the beam moves away from the center, detector calibration is necessary. Calibration is performed by measuring the response of the QD as a laser beam is traversing the sensor. Here, we assume that the response of the QD is the same for a beam traversing along the x axis and along the y axis. QD response varies with beam diameter. As such, we performed the calibration on different values of beam diameter.

The QD used in our measurements, Thorlabs PDQ80A, has a sensor size of 7.8 mm, hence we varied the beam diameter so as not to exceed half of the QD sensor size. Fig S6a shows the QD response as the incident beam traverses the x axis of the QD at different beam diameter values. Zero values of the response indicates that the incident beam is at the center of the QD. The plot in Fig. S6a have been adjusted to reflect this. The linear trend in the center of the QD is ideal for a typical QD. The curved part indicates that the beam is already reaching the edge of the QD segments. The calibration constant for each beam diameter is obtained from the slope of the linear part. The dependence of the slope and the calibration constant to the beam diameter is shown in Figs. S3b-c. As shown in Fig. S3c, the calibration constant increases linearly with the beam diameter as such via linear regression, we can easily extract the calibration constant for any beam diameter.

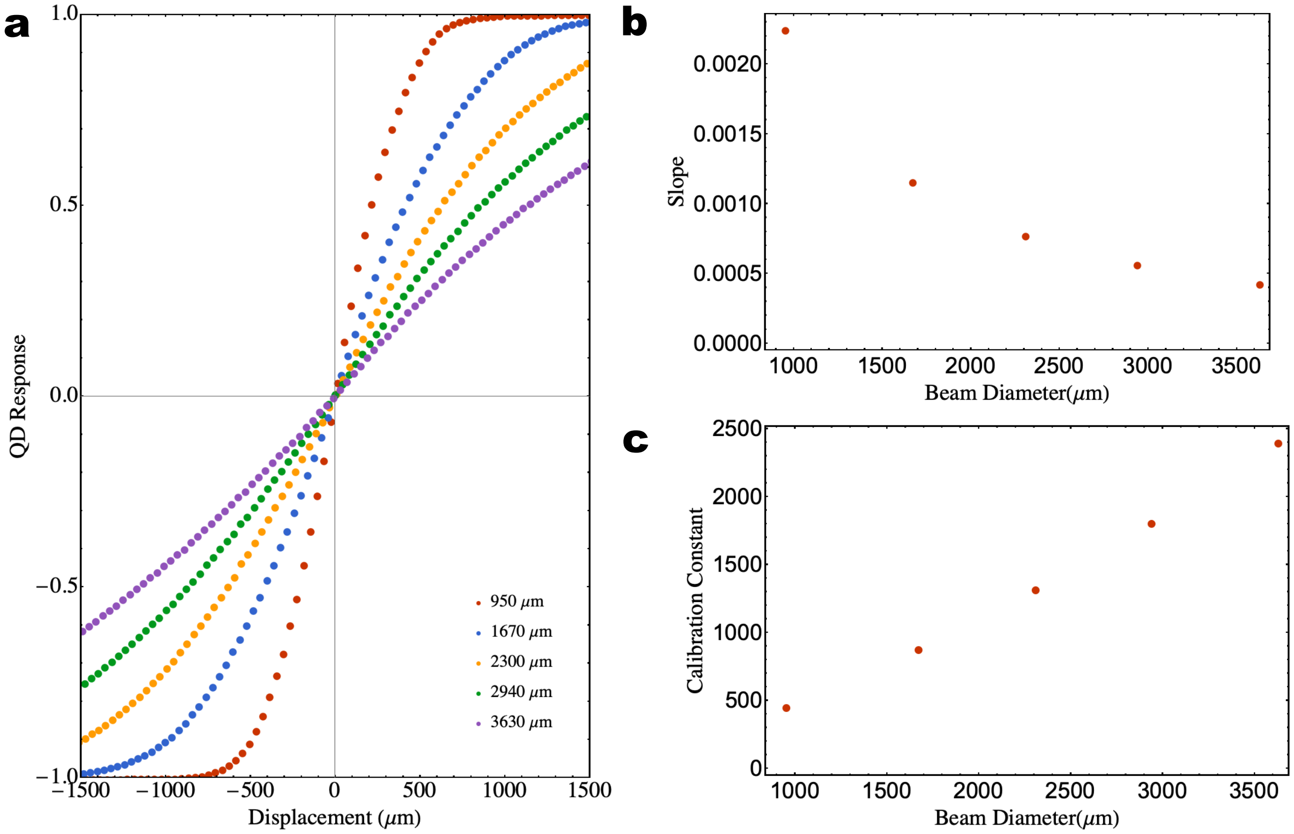

Figure S6: (a) Beam diameter dependence of QD response as the incident beam traverses across the active region of the QD. (b) Slope of the linear part in (a) for different beam diameter values. (c) Corresponding calibration constants of the QD for different beam diameters. Linear regression from this plot was used to obtain the calibration constant of the incident beam used in the measurements.

References

[1] Z. Qin, Q. Liu, C. Liu, C. Yue, and Y. Lang, "Enhanced in-plane and out-of-plane photonic spin Hall effect via surface plasmon resonance," *Physics of Plasmas*, vol. 25, no. 2, 2018.

[2] X. Jiang, Q. Wang, J. Guo, S. Chen, X. Dai, and Y. Xiang, "Enhanced photonic spin Hall effect with a bimetallic film surface plasmon resonance," *Plasmonics*, vol. 13, pp. 1467–1473, 2018.

[3] H. Luo, X. Zhou, W. Shu, S. Wen, and D. Fan, "Enhanced and switchable spin Hall effect of light near the Brewster angle on reflection," *Physical Review A*, vol. 84, no. 4, p. 043806, 2011.

[4] M.-M. Pan, Y. Li, J.-L. Ren, B. Wang, Y.-F. Xiao, H. Yang, and Q. Gong, "Impact of in-plane spread of wave vectors on spin Hall effect of light around Brewster's angle," *Applied Physics Letters*, vol. 103, no. 7, 2013.

[5] J.-L. Ren, B. Wang, M.-M. Pan, Y.-F. Xiao, Q. Gong, and Y. Li, "Spin separations in the spin Hall effect of light," *Physical Review A*, vol. 92, no. 1, p. 013839, 2015.

[6] Z. Qin, Q. Liu, C. Yue, and Y. Lang, "Modified model of photonic spin hall effect of gaussian beam reflected from a dielectric interface," *Applied Physics Express*, vol. 12, no. 6, p. 062008, 2019.

[7] K. Y. Bliokh and A. Aiello, "Goos–Hänchen and Imbert–Fedorov beam shifts: an overview," *Journal of Optics*, vol. 15, no. 1, p. 014001, 2013.

[8] X. Zhou and X. Ling, "Enhanced photonic spin Hall effect due to surface plasmon resonance," *IEEE Photonics Journal*, vol. 8, no. 1, pp. 1–8, 2016.

[9] A. Aiello and J. Woerdman, "Role of beam propagation in Goos–Hänchen and Imbert–Fedorov shifts," *Optics letters*, vol. 33, no. 13, pp. 1437–1439, 2008.
